# Geographical Variation in Bacterial Community Diversity and Composition of *Corythucha ciliata*

**DOI:** 10.3390/microorganisms13122748

**Published:** 2025-12-02

**Authors:** Tong-Pu Li, Hao-Xin Li, Jia-Sheng Bao, Chen-Hao Wang, Kai-Lu Wang, Bing-Ren Hao, Zhi-Heng Wang, Jia-Hui Hu, Lv-Quan Zhao

**Affiliations:** Co-Innovation Center for Sustainable Forestry in Southern China, College of Forestry and Grassland, Nanjing Forestry University, Nanjing 210037, China; tpli@njfu.edu.cn (T.-P.L.);

**Keywords:** *Corythucha ciliata*, bacterial community, geographical differentiation, climate factors, functional traits, invasive adaptation

## Abstract

The sycamore lace bug, *Corythucha ciliata*, a globally invasive pest that damages *Platanus* spp., harbors a bacterial microbiome that may help it adapt to different geographical environments. However, the geographical differentiation patterns of its bacterial community and the underlying driving mechanisms remain unclear. In this study, we standardized rearing of three *C. ciliata* populations (collected from Beijing, Lianyungang, and Nanjing) for three generations to reduce immediate environmental interference, then analyzed their bacterial communities via *16S rRNA* gene amplicon sequencing. The principal coordinate analysis revealed a significant separation of the bacterial community in the Nanjing population, while the Beijing and Lianyungang populations were more similar. Bacterial alpha diversity followed the gradient of “Nanjing > Lianyungang > Beijing”, with the Nanjing population exhibiting significantly higher species richness and evenness than the Beijing population. All three populations shared core bacterial taxa (e.g., phyla Proteobacteria, Bacteroidota; genera *Cardinium*, *Serratia*), but their relative abundances differed significantly: *Cardinium* dominated the Beijing population (50.1%), *Serratia* dominated the Lianyungang population (45.86%), and the Nanjing population harbored unique dominant genera such as *Sphingomonas*. For the three target populations, monthly average temperature and wind speed were positively correlated with bacterial diversity, while latitude was negatively correlated (Pearson correlation coefficient: 0.6564 < |*r*| < 0.7010, *p* < 0.05). Core bacterial functions (e.g., substance transport) were conserved across populations, whereas differential functions (e.g., detoxification, lipid metabolism) were linked to geographical adaptation. This study confirms the climate-driven geographical differentiation of the *C. ciliata* bacterial community provides insights into the “insect–microbiome” interactive invasion mechanism that is present here.

## 1. Introduction

The sycamore lace bug, *Corythucha ciliata* (Hemiptera: Tingidae), a globally invasive pest, illustrates the interplay between geography and ecological adaptation. Native to temperate eastern North America, this species has invaded Eurasia through dual pathways since the 1960s: one spreading via Europe to inland China, and the other colonizing eastern coastal regions through the Korean and Japanese peninsulas, ultimately establishing overlapping distribution zones in East Asia [1,2]. Currently, its distribution covers more than 20 provinces (e.g., Jiangsu, Shandong, Hubei, Beijing) in China, causing severe damage to *Platanus* spp. species which are key urban greening trees [3,4,5]. *C. ciliata* completes 2–5 annual generations, with overwintering adults sheltering in bark crevices by late October in subtropical regions like Nanjing. Upon resuming activity in April, population densities reach 150 individuals per tree, causing > 60% leaf chlorosis [6,7,8,9,10]. Genomic studies reveal adaptations to thermal stress via redox enzyme gene family expansion and phosphorylation-mediated regulation of HSP70/90 [2]. However, the synergistic role of the microbiome in its invasion process has not been systematically studied, and this constitutes a critical gap in understanding the mechanisms underlying its wide-ranging distribution.

The symbiotic relationship between insects and microorganisms represents a core evolutionary strategy for species to adapt to their environments, and this relationship is particularly prominent in herbivorous insects [11,12,13,14,15]. The primary symbiont *Buchnera aphidicola* assists aphids in synthesizing essential amino acids, compensating for the nutritional deficiencies in the phloem sap of host plants [16,17]. In extreme environments, this symbiotic relationship is further strengthened. High-altitude bumblebees enhance energy supply through butyrate metabolism by the gut bacterium *Snodgrassella*, and this metabolic process enables their survival in low-temperature and hypoxic conditions [18]. More complex symbiotic networks manifest in functional complementarity. For instance, the facultative symbiont *Serratia* in aphids can encode the DNA mismatch repair protein MutH [19]. This protein compensates for the genetic defects of *Buchnera* and significantly improves the host aphids’ heat tolerance. In the field of invasion biology, the plasticity of the microbiome has been shown to be important for the rapid adaptation of species: the Colorado potato beetle *Leptinotarsa decemlineata* decomposes mandelonitrile produced by its symbiont *Proteus vulgaris* into hydrogen cyanide to form a chemical defense barrier [20]; the fall webworm *Hyphantria cunea* acquires the bacterial chitinase gene HcuChiA through horizontal gene transfer, and this gene is specifically expressed in the gut to enhance the insect’s adaptation to *Metasequoia* [21]. Collectively, these studies demonstrate that the microbiome is not only a metabolic organ of the host but also an active regulatory module for its environmental adaptation.

Despite significant progress in insect microbiome research, studies on the microbiome of *C. ciliata* still have obvious limitations. Methodologically, most insect microbiome studies fail to strictly control the host’s genetic background, making it difficult to distinguish the independent effects of genetic factors and environmental filtering [22]. For example, a recent study on pollinators found that ecological and physiological factors play important roles in determining the structure and function of microbial communities, and there is significant differentiation in microbiomes among different taxa [23]. In terms of research subjects, the symbiont composition of hemipteran species such as the Asian citrus psyllid *Diaphorina citri* and the small brown planthopper is significantly correlated with latitude and annual temperature [24,25]. Within the Tingidae family, however, only the pear lace bug *Stephanitis nashi* has been studied in this regard. Its internal microbiome is dominated by *Wolbachia* and *Rickettsia*, and is significantly influenced by the host plant species [26,27]. In contrast, research on the microbiome of *C. ciliata* has three key gaps: the first is that the characteristics of its core microbial community remain unknown; the second is that the geographic differentiation patterns of its microbiome are unclear; and the third is that the functional potential of its microbiome has not been explored. Additionally, current control strategies rely excessively on chemical methods, neglecting the regulatory potential of the microbiome in stress resistance.

Therefore, this study aims to systematically analyze the geographic differentiation patterns of the *C. ciliata* microbiome and its functional mechanisms in invasion adaptation. Three representative geographic populations from Beijing, Lianyungang, and Nanjing were selected, and three generations of standardized rearing were conducted to minimize immediate environmental interference. Combined with *16S rRNA* gene amplicon sequencing technology, the composition, geographic variation, and functional adaptability of the *C. ciliata* microbiome were systematically analyzed. The key research objectives are: (1) to characterize the core microbial community and its environmental driving mechanisms; (2) to explore the association between microbial functional modules and host stress resistance; (3) to construct a microbe-mediated ecological adaptation model. The results of this study will provide new targets for the green control of invasive species and promote the in-depth development of the “insect–microbiome” co-adaptation theory.

## 2. Materials and Methods

### 2.1. Collection and Rearing of Test Insects

To investigate the shaping effect of different geographical populations of *C. ciliata* on their microbial communities, three *C. ciliata* populations were collected from the abaxial surface of sycamore leaves, respectively, in Beijing (39° N), Lianyungang (35° N), and Nanjing (31° N) in August 2024. To minimize the interference of geographical and climatic factors on the insect microbial communities and highlight the influence of population background, the three *C. ciliata* populations were continuously reared for three generations under identical laboratory conditions. The specific rearing method was as follows: *C. ciliata* individuals were brought back to the laboratory together with their host leaves and reared in an artificial climate chamber under standardized conditions (temperature: 26 ± 0.5 °C, relative humidity: 80 ± 5%). The rearing containers were transparent plastic boxes with dimensions of 25.3 cm × 17.3 cm × 9.3 cm, and dense ventilation holes were made on the box lids using No. 4 insect needles. A 2 cm-thick sponge saturated with water was placed at the bottom of each box; fresh sycamore leaves were laid flat on the sponge surface with their abaxial sides facing upward, and then the lace bugs were transferred onto the leaves. Leaves were replaced and water was replenished every 7 days, maintaining the water level at half the height of the sponge. During leaf replacement, adults and nymphs were transferred using a small brush, while dead individuals and excreta were removed simultaneously to keep the rearing environment clean. The rearing conditions and food supply were kept consistent across the three *C. ciliata* populations to ensure the normal survival and reproduction of adults.

### 2.2. Collection, Sequencing, and Data Assembly of Microbial Samples

After the three *C. ciliata* populations were continuously reared and propagated for 3 generations under the same environment, the sample collection, sequencing, and data assembly were carried out. Referring to previous study, each *C. ciliata* population was set with 3 biological replicates, and 5 adult bodies were sampled per replicate (adult gender was not distinguished during sampling), resulting in a total of 9 samples [28]. After collection, the surface of each insect was wiped with 75% ethanol for 90 s to remove attached microorganisms, followed by rinsing with sterile deionized water 3 times to completely minimize residual ethanol and contaminants.

Genomic DNA of the samples was extracted using the DNeasy^®^ Blood & Tissue DNA Extraction Kit (Qiagen, Düsseldorf, Germany). The V3–V4 hypervariable regions of the bacterial *16S rRNA* gene were targeted for PCR amplification using specific primers (forward primer: 5′-CCTAYGGGRBGCASCAG-3′; reverse primer: 5′-GGACTACNNGGGTATCTAAT-3′) [29]. The PCR products were separated by 2% agarose gel electrophoresis and purified using the AxyPrep DNA Gel Extraction Kit (Axygen Biosciences, a brand of Corning Inc., Corning, NY, USA). After quantification with Qubit 3.0 Fluorometer (v3.0, Thermo Fisher Scientific Inc., Waltham, MA, USA), a PE250 (2 × 250 bp paired-end) sequencing library was constructed, and high-throughput sequencing was finally performed on the Illumina NovaSeq 6000 platform (Illumina Inc., San Diego, CA, USA).

Raw data were distinguished by index sequences and stored in FASTQ format. The processing workflow of sequencing data referred to previous studies [30,31]. Simply, the DADA2 plugin (v1.26.0) within QIIME 2 (v2023.9, QIIME 2 Development Team, University of Colorado Boulder, Boulder, CO, USA) was used for sequence quality filtering (removal of sequences with length < 200 bp or average quality score < 20), denoising, and removal of chimeric sequences to directly generate amplicon sequence variants (ASVs). Singleton ASVs were further removed to reduce noise interference. The optimized representative sequences of ASVs were aligned against the Silva database (v138.1, Quast C et al., Technical University of Munich, Munich, Germany) to generate an ASV abundance table.

### 2.3. Microbial Community Analysis

Alpha diversity analysis was performed using QIIME 2 to calculate the species richness (Chao1 index, ACE index) and evenness (Shannon index, Simpson index) of the three *C. ciliata* populations, so as to characterize the bacterial community diversity of different populations; the Chao1 index and Shannon index were selected for visual presentation. In beta diversity analysis, principal coordinates analysis (PCoA) was performed for visualization based on Bray–Curtis distances, and Permutational Multivariate Analysis of Variance (PERMANOVA) was used to test the significance of differences in microbial communities among populations. In addition, based on the relative abundances of ASVs at the phylum and genus levels, stacked bar charts of bacterial community structures were plotted for each sample (genera with relative abundance ranking beyond the top 15 were merged into “others” to simplify the presentation). Meanwhile, Circos plots (using circlize package, v0.4.15, within R software) and Triangle plots (using vegan package, v2.6-4, within R software) were used to intuitively display the corresponding relationships between samples of each population and microorganisms at the phylum/genus level.

### 2.4. Correlation Analysis of Geographical and Climatic Factors

To clarify the association between the bacterial communities of *C. ciliata* and geographical climatic factors, the linear correlations between bacterial diversity indices (Chao1 index, Shannon index), and climatic factors were analyzed. Data on monthly average temperature and monthly average wind speed in August from 2008 to 2024 (17 years) for Beijing, Lianyungang, and Nanjing were obtained from sources including the National Centers for Environmental Information (NCEI, Asheville, NC, USA) and local government statistical yearbooks. Linear regression analysis was then performed using the lm function within R software (v4.3.2, R Foundation for Statistical Computing, Vienna, Austria) between the above climatic data and the Chao1 index, Shannon index of bacterial communities, as well as the relative abundances of Proteobacteria and Bacteroidota in the three populations.

### 2.5. Prediction of Microbial Functions

To explore the influence of different geographical *C. ciliata* populations on the functions of their microbial communities, functional prediction of microorganisms in the three geographical populations was conducted. First, taxonomic annotation and abundance quantification were performed on the *16S rRNA* gene amplicon data obtained from raw sequencing. Subsequently, functional genomics prediction analysis was carried out using the PICRUSt2 platform (v2.5.2, Harvard T.H. Chan School of Public Health, Boston, MA, USA): phylogenetic information from reference genome databases (including the Integrated Microbial Genomes database, v5.0, Joint Genome Institute, Walnut Creek, CA, USA and EBI Metagenomics database, European Bioinformatics Institute, Cambridge, UK) was integrated to construct a species-function mapping profile. Then, the Weighted Nearest Sequenced Taxon Index (WNSTI) algorithm was used to predict the abundance of KEGG orthology (KO) functional gene families.

### 2.6. Data Processing and Statistical Analysis

To ensure the statistical reliability of experimental data, the Shapiro–Wilk test and Levene test were used to conduct normality test and homogeneity of variance test, respectively, on data including Goods’ coverage, alpha diversity indices, bacterial relative abundances, and microbial KO functional abundances of the three *C. ciliata* populations. For data that conformed to normal distribution and homogeneous variance, one-way Analysis of Variance (one-way ANOVA) combined with post hoc least significant difference (LSD) method was used for pairwise comparisons; for data that did not meet the above conditions, the Kruskal–Wallis test combined with post hoc Dunn’s method was used for pairwise comparisons. Based on Bray–Curtis distances, PERMANOVA was used to evaluate the differences in beta diversity of microbial communities among populations. Pearson correlation coefficient was used to test linear relationships, so as to assess the explanatory power of environmental factors on the variation of microbial diversity. All statistical analyses were performed using R software (v4.3.2, R Foundation for Statistical Computing, Vienna, Austria). For result visualization and supplementary statistical verification to ensure result consistency, GraphPad Prism software (v9.5.1, GraphPad Software Inc., San Diego, CA, USA) was additionally used when necessary.

## 3. Results

### 3.1. Sequencing Data Quality Profile

**Figure 1:**
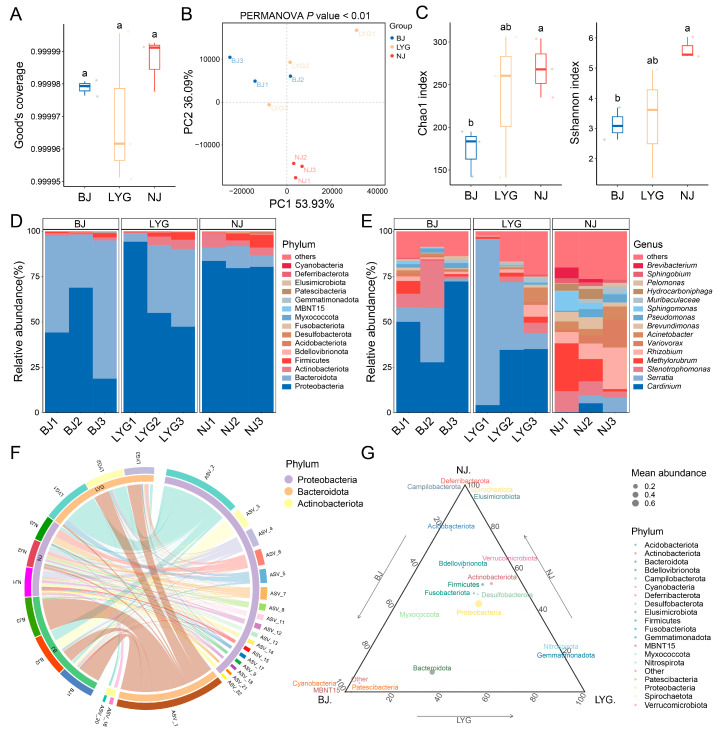
Comparison of bacterial diversity and structural composition among three *C. ciliata* populations. (**A**) Goods’ coverage of the three *C. ciliata* populations. (**B**) Principal coordinate analysis showing beta diversity patterns among the three *C. ciliata* populations. (**C**) Alpha diversity metrics, including bacterial community richness (Chao1 index) and species diversity (Shannon index) across the three *C. ciliata* populations. Different letters indicate significant differences. (**D**,**E**) Structural composition of bacterial communities at the phylum (**D**) and genus (**E**) levels, displaying the top 15 predominant phyla and genera, with remaining taxa grouped as “others”. (**F**) Circos plots illustrating the correspondence between samples and bacterial abundances of the top 3 predominant phyla. (**G**) Triangle plot showing the distribution of predominant bacterial phyla across the three *C. ciliata* populations. BJ, Beijing population; LYG, Lianyungang population; NJ, Nanjing population.

Quality control and evaluation were conducted on the *16S rRNA* gene sequencing data of *C. ciliata* populations from three locations: Beijing (BJ), Lianyungang (LYG), and Nanjing (NJ). The results showed that the Good’s coverage of all samples reached 99.99%, indicating near-complete coverage of the microorganisms in the community and ensuring extremely high integrity and reliability of the sequencing data (Table 1, Figure 1A). A total of 645,699 reads were obtained from this sequencing, with an average of 71,744.33 reads per sample. Specifically, 217,426 reads were obtained from the BJ population, 208,871 reads from the NJ population, and 219,402 reads from the LYG population. Through *16S rRNA* gene sequencing analysis, a total of 1782 ASVs were identified in the bacterial microbiomes of the three *C. ciliata* populations, which were classified into 22 phyla, 44 classes, 117 orders, 187 families, and 329 genera.

### 3.2. Bacterial Community Diversity of the Three Populations

To clarify the effect of population background on the bacterial community of *C. ciliata*, beta diversity patterns of the bacterial communities among the three populations were characterized (Figure 1B). Principal coordinates analysis showed a significant separation trend in the bacterial communities of the three populations. The first two principal coordinates (PC1 and PC2) together explained 74.25% of the variation in the bacterial communities, with PC1 accounting for 53.93% and PC2 for 20.32%. From the sample clustering pattern: the BJ population was concentrated in the left region of PC1, while the LYG and NJ populations were mainly clustered in the right region of PC1; meanwhile, the BJ and LYG populations were concentrated in the right region of PC2, and the NJ population was mainly clustered in the left region of PC2. Further PERMANOVA analysis confirmed that there were extremely significant differences in bacterial communities among different populations (PERMANOVA, *p* < 0.01). These results indicated that geographical distribution was a key factor leading to the differentiation of *C. ciliata* bacterial communities, and the communities of the BJ and LYG populations showed higher similarity, while the community difference between these two populations and the NJ population was more significant.

Alpha diversity indices of the three *C. ciliata* populations exhibited a gradient pattern, with an overall trend of “NJ > LYG > BJ” (Table 1, Figure 1C). In terms of species richness: the NJ population had the highest Chao1 index (269.04 ± 34.53) and the highest ACE index (270.9 ± 33.83) simultaneously; the LYG population ranked second (Chao1 index: 235.92 ± 85.04; ACE index: 235.95 ± 84.64); and the BJ population had the lowest values (Chao1 index: 173.46 ± 27.85; ACE index: 173.83 ± 28.33). In terms of species evenness: the Shannon index (5.63 ± 0.35) and Simpson index (0.95 ± 0.02) of the NJ population were significantly higher than those of the LYG population (Shannon index: 3.31 ± 1.8; Simpson index: 0.68 ± 0.25) and the BJ population (Shannon index: 3.14 ± 0.53; Simpson index: 0.67 ± 0.17), while there was no significant difference between the latter two populations. Statistically, both the species richness (Chao1 index) and evenness (Shannon index) of the NJ population were significantly higher than those of the BJ population (one-way ANOVA with post hoc LSD test, *p* < 0.05), while the LYG population was between the two and showed no significant statistical difference from them. These results indicated that the *C. ciliata* population in Nanjing had the highest Alpha diversity of bacterial communities (rich species with uniform distribution), the BJ population had the lowest, and the LYG population was in between, preliminarily revealing the significant impact of geographical factors on the bacterial community diversity of populations.

### 3.3. Bacterial Community Structure of the Three Populations

**Figure 2:**
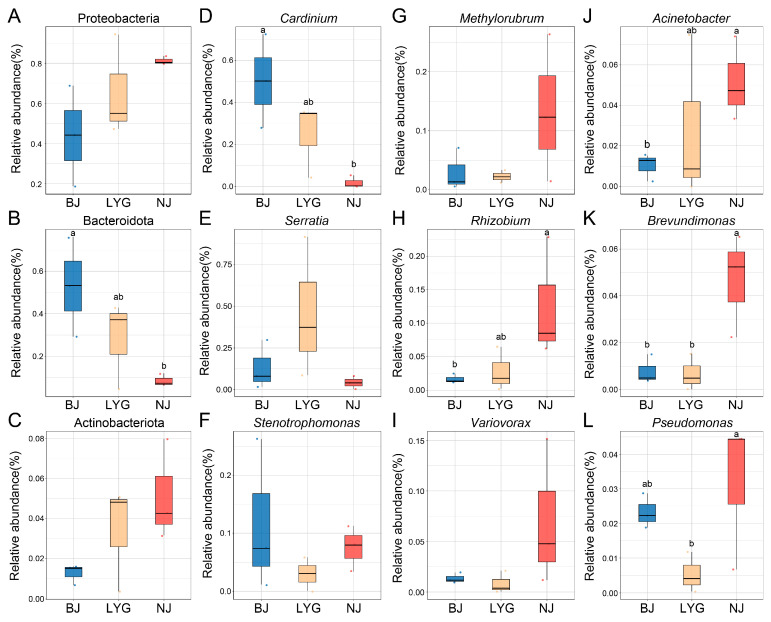
Comparative analysis of bacterial community differentiation among three *C. ciliata* populations. (**A**–**C**) Comparisons of relative abundances of key bacterial phyla: Proteobacteria (**A**), Bacteroidota (**B**) and Actinobacteriota (**C**). (**D**–**L**) Comparisons of relative abundances of key bacterial genera: *Cardinium* (**D**), *Serratia* (**E**), *Stenotrophomonas* (**F**), *Methylorubrum* (**G**), *Rhizobium* (**H**), *Variovorax* (**I**), *Acinetobacter* (**J**), *Brevundimonas* (**K**), and *Pseudomonas* (**L**). Different letters indicate significant differences among populations (BJ, LYG, NJ). BJ, Beijing population; LYG, Lianyungang population; NJ, Nanjing population.

To clarify the specific compositional differences in bacterial communities among the three populations and analyze the shaping effect of different geographical environments on community structure, the community structure characteristics were analyzed at both phylum and genus levels. At the phylum level, the three populations shared the same core dominant phyla, namely Proteobacteria, Bacteroidota, and Actinobacteriota, but their relative abundances differed (Figure 1D,F,G; Figure 2A–C). As the most dominant phylum, Proteobacteria showed a relative abundance trend of “NJ (81.25%) > LYG (65.55%) > BJ (43.96%)”. Bacteroidota, as the second dominant phylum, showed a relative abundance pattern of “BJ (52.93%) > LYG (28.30%) > NJ (8.58%)”, and there was a significant difference in the relative abundance of Bacteroidota between the BJ and NJ populations (one-way ANOVA with post hoc LSD test, *p* < 0.05). The relative abundance of Actinobacteriota was relatively low, but the NJ population (5.13%) had a significantly higher abundance than the LYG population (3.42%) and the BJ population (1.26%). At the genus level, the three populations shared core dominant genera including *Cardinium*, *Serratia*, and *Stenotrophomonas*, but their relative abundances differed significantly (Figure 1E). *Cardinium* had the highest abundance in the BJ population (50.1%), while *Serratia* had the highest abundance in the LYG population (45.86%). In addition, the NJ population also had unique dominant genera such as *Sphingomonas* and *Acinetobacter*, and the abundance of *Sphingomonas* in the NJ population was significantly higher than that in the BJ and LYG populations. These results indicated that the core bacterial taxa of the three populations were consistent, but there were significant differences in relative abundances at both phylum and genus levels; moreover, the NJ population had higher genus-level diversity, further reflecting the selective shaping of community structure by geographical environmental background.

This study further compared the relative abundances of 9 key dominant bacterial genera at the genus level among the three *C. ciliata* populations (Figure 2D–L). The results showed that except for *Cardinium*, the NJ population exhibited a significant enrichment effect for most genera such as *Rhizobium*, *Acinetobacter*, *Brevundimonas*, and *Pseudomonas*, with their abundances being significantly higher than those in the BJ or LYG populations. Only the relative abundance of *Methylorubrum* in the BJ population was significantly higher than that in the LYG and NJ populations (one-way ANOVA with post hoc LSD test, *p* < 0.05). The LYG population was in a “transitional state” between the BJ and NJ populations in terms of the abundance of most genera. In contrast, the abundances of *Stenotrophomonas* and *Variovorax* showed no significant differences among the three populations. Overall, these results suggested that environmental conditions associated with geographical latitude might be the key factor driving the differentiation of bacterial communities at the genus level.

### 3.4. Correlation Between Bacterial Community Characteristics and Geographical-Climatic Factors

**Figure 3:**
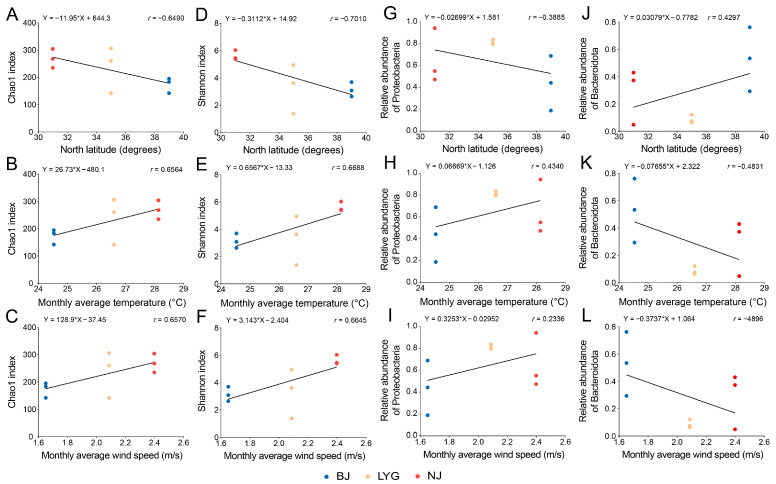
Correlations between bacterial community characteristics of *C. ciliata* and environmental factors. (**A**–**C**) Linear regression analysis of bacterial Chao1 index with north latitude (**A**), monthly average temperature (**B**), and monthly average wind speed (**C**). (**D**–**F**) Linear regression analysis of bacterial Shannon index with north latitude (**D**), monthly average temperature (**E**), and monthly average wind speed (**F**). (**G**–**I**) Linear regression analysis of Proteobacteria relative abundance with north latitude (**G**), monthly average temperature (**H**), and monthly average wind speed (**I**). (**J**–**L**) Linear regression analysis of Bacteroidota relative abundance with north latitude (**J**), monthly average temperature (**K**), and monthly average wind speed (**L**). Each dot represents a sample from different *C. ciliata* populations: blue (BJ, Beijing population), orange (LYG, Lianyungang population), red (NJ, Nanjing population). The equation and correlation coefficient (*r*) for each linear relationship are shown in the corresponding subplot.

To clarify the regulatory effect of geographical-climatic factors on the bacterial community characteristics of *C. ciliata*, this study analyzed the environmental driving factors of differences in bacterial community diversity and structure (Figure 3). Alpha diversity indices were closely correlated with climatic factors: both the Chao1 index (species richness) and Shannon index (species evenness) were significantly positively correlated with monthly average temperature (Pearson correlation test, *r* = 0.6564 and 0.6688, respectively, *p* < 0.05) and monthly average wind speed (*r* = 0.6570 and 0.6645, respectively, *p* < 0.05), while they were significantly negatively correlated with north latitude (*r* = −0.6490 and −0.7010, respectively, *p* < 0.05). These results indicated that temperature and wind speed were the dominant factors affecting the bacterial community characteristics of the three populations, and higher temperature and wind speed could promote the improvement of community diversity. Latitude difference is precisely the core reason for the spatial heterogeneity of temperature and wind speed. This result was completely consistent with the characteristics that the NJ population had the highest bacterial diversity and the BJ population had the lowest, clarifying the regulatory mechanism of geographical-climatic factors on community characteristics.

### 3.5. Functional Prediction Analysis of Bacterial Communities in the Three Populations

To explore the differences in functional potential of bacterial communities among different populations, the conservation of core functions, and the association between bacterial functions and the geographical adaptability of hosts, functional prediction of bacterial communities was performed (Table 2). The results showed that the top 10 high-abundance KO functional categories in terms of abundance were highly consistent among the three populations, mainly involving substance transport (e.g., ABC transport system-related proteins, iron complex outer membrane receptor proteins), transcriptional regulation (e.g., LacI family transcriptional regulators), and stress response (e.g., cold shock protein CspA family). Most of these functions showed no significant differences among populations, indicating that the core functions of the *C. ciliata* bacterial community exhibited strong conservation and were less affected by geographical distribution. In terms of differential functions, the functions with significant abundance differences were mainly concentrated in the fields of detoxification, metabolism, and stress response: the abundances of glutathione S-transferase (detoxification-related enzyme) and 3-oxoacyl-[acyl-carrier protein] reductase (lipid metabolism-related) in the NJ population were significantly (*p* < 0.05) or extremely significantly (*p* < 0.01) higher than those in the BJ and LYG populations; the abundance of cold shock protein in the BJ population was significantly lower than that in the LYG and NJ populations; and the abundances of related functions in the LYG population were mostly between those in the BJ and NJ populations. These results indicated that the core functions of the *C. ciliata* bacterial community were conserved, while the differential functions were mainly related to the environmental adaptability of the host—among which the detoxification and metabolic functions of the NJ population were more active, further reflecting the selective shaping effect of geographical environment on bacterial functions.

## 4. Discussion

In this study, three geographical populations of *C. ciliata* from Beijing, Lianyungang, and Nanjing were subjected to standardized rearing for three generations. Combined with *16S rRNA* gene amplicon sequencing, we systematically investigated the geographical differentiation pattern of their bacterial communities, with the core objective of clarifying the “geography–climate–microbiome” linkage and the role of the microbiome in invasive adaptation. The results showed that the bacterial alpha diversity exhibited a geographical gradient of “NJ > LYG > BJ”, where the species richness and evenness of the Nanjing population were significantly higher than those of the Beijing population. Beta diversity analysis revealed significant differentiation among the three populations: the Beijing and Lianyungang populations had higher similarity, while the Nanjing population was clearly separated. In terms of community structure, the three populations shared core dominant phyla such as Proteobacteria, Bacteroidetes, and Actinobacteria, as well as core dominant genera including *Cardinium* and *Serratia*, but with significant differences in relative abundance. For instance, *Cardinium* had the highest abundance in the Beijing population, *Serratia* was dominant in the Lianyungang population, and the Nanjing population possessed unique dominant genera such as *Sphingomonas*. Climatic factors (monthly average temperature, wind speed) were positively correlated with bacterial diversity, though additional variables (e.g., host plant traits) may also play roles, while latitude was negatively correlated with diversity. Functionally, core functions (e.g., substance transport, transcriptional regulation) were conserved, and differential functions (e.g., detoxification, lipid metabolism) were associated with geographical adaptation. These findings fill the research gap in the geographical variation of the *C. ciliata* microbiome, provide a new perspective for understanding the “host–microbiome” interactive invasion mechanism, and confirm the driving role of geographical climate in microbiome differentiation and the dual value of the microbiome in invasive adaptation.

The geographical differentiation of bacterial diversity in *C. ciliata* reflects the host’s regional adaptation strategy, with the core being the “low latitude-high microbial diversity” pattern and the dominant role of environmental isolation in community differentiation. The subtropical climate in Nanjing (31° N), characterized by high temperature (28.03 °C in August) and high wind speed (2.43 m/s), may have promoted the increase in microbial diversity by enhancing the metabolic activity of the host and its symbiotic microorganisms, as well as expanding the sources of microbial colonization (e.g., wind dispersal) [32,33,34]. Similarly, a study on *D. citri* found that the gut microbial diversity of low-latitude populations was higher than that of high-latitude populations, which was mainly attributed to the regulation of community assembly by temperature [25]. In contrast, among the significant differentiation of beta diversity, the Beijing and Lianyungang populations had overlapping climate zones (temperate to warm temperate), resulting in low barriers to microbial gene flow and high community similarity. The Nanjing population, however, was far from Beijing (approximately 1000 km) and had distinct subtropical climate characteristics, imposing stronger selection pressure on the microbiome [34,35]. It is thus evident, that in invasive species, the driving effect of environmental differences on microbial differentiation is stronger than that of geographical distance [23,36,37]. These findings suggest that the geographical differentiation of bacterial diversity in *C. ciliata* is not solely caused by geographical distance, but rather a direct reflection of the host’s regional adaptation strategy dominated by climatic heterogeneity.

The core driver of the structural differentiation of the *C. ciliata* microbial community lies in the dual mechanism whereby conserved core microbial groups ensure survival and environment-specific differential groups adapt to local conditions. The three populations shared core taxa such as Proteobacteria (abundance: 43.96–81.25%), Bacteroidetes, Actinobacteria, *Cardinium*, and *Serratia*, which reflects the evolutionarily conserved essential symbiotic relationships [38,39]. Proteobacteria is a core component of the gut microbiome in hemipteran insects; for example, the proteobacterial symbiont *B. aphidicola* in aphids supplements essential amino acids for the host. Thus, Proteobacteria in *C. ciliata* may also provide nutrients by decomposing secondary metabolites in sycamore leaves [16,17,40,41]. Meanwhile, *Cardinium*, as a dominant genus with an abundance of 50.1% in the Beijing population, may enhance the insect’s cold tolerance by upregulating antifreeze protein genes, adapting to the cold winters in Beijing and thereby complementing the host’s own heat stress adaptation [21,42,43,44]. Therefore, the core microbial groups form the basis for the cross-geographical survival of *C. ciliata*, and their conservation ensures the fulfillment of the host’s basic physiological needs.

Although the core microbial groups are conserved, the genus-level differential taxa represent adaptive adjustments of the microbiome to the local environment. *Serratia*, with an abundance of 45.86% in the Lianyungang population, possesses detoxification capabilities for degrading allelochemicals such as plant phenolics [20,45]. As a coastal city, Lianyungang has high humidity (75–80% in August) and frequent plant diseases, which lead to an increase in defensive secondary metabolites in sycamore leaves. The enrichment of *Serratia* may help the host cope with this chemical stress [46,47]. The *Sphingomonas* genus, unique to the Nanjing population, had a significantly higher abundance than in the other populations; this genus can assist the host in cellulose metabolism and exhibits heat resistance, adapting to the demand for efficient nutrient utilization under the high temperature and long growing season in Nanjing [26,48]. Additionally, the enriched *Acinetobacter* genus in the Nanjing population may be associated with insect lipid metabolism, providing energy reserves for its rapid expansion, thus endowing the Nanjing population with a greater reproductive advantage compared to the Beijing population (4–5 generations per year in Nanjing vs. 2–3 generations per year in Beijing) [27,49]. In summary, the differential microbial groups serve as adaptive tools for *C. ciliata* to cope with stress in different geographical environments, directly enhancing the host’s survival competitiveness in local habitats.

Microbial functional traits of *C. ciliata* follow the same “conserved-differential” logic to support host invasive adaptation: conserved functions guarantee basic survival, while differential ones enhance local adaptation. Functional prediction revealed that the core functions of the three populations (e.g., ABC transport system, LacI family transcriptional regulators, cold shock proteins) were highly conserved. For example, the ABC transport system is associated with the absorption of nutrients such as amino acids and carbohydrates; LacI family regulators can respond to nutrient fluctuations; and cold shock proteins can protect cells from temperature-induced damage. These functions ensure the stability of the host’s basic physiological activities in different geographical environments and represent common characteristics of the symbiotic systems of invasive species [15,17,19,21]. In contrast, differential functions are specifically adapted to local conditions: the Nanjing population had a significantly higher abundance of glutathione S-transferase (GST, a detoxifying enzyme), which can bind to toxic substances to reduce their harm, adapting to the environment of sycamore leaves with high levels of flavonoids and tannins under the warm climate in Nanjing [6,21]. The Beijing population had a lower abundance of cold shock proteins, possibly because the host has evolved low-temperature adaptation mechanisms such as the expansion of the oxidoreductase gene family, and instead maximizes energy intake by decomposing complex organic matter through Bacteroidetes [2,18,50]. Therefore, the conservation of microbial functions provides a fundamental guarantee for the invasion of *C. ciliata*, while the differentiation enhances its adaptability to different geographical environments; the two work synergistically to improve the host’s invasive ability.

## 5. Conclusions

In conclusion, this study focused on the geographical differentiation of the *C. ciliata* microbiome, with the core objective of clarifying the microbiome’s role in invasive adaptation. The results suggest that the *C. ciliata* microbiome exhibits a climate-driven geographical differentiation pattern, forming an “insect–microbiome” co-adaptive system together with host genomic adaptation, and show that conserved core groups likely ensure survival, while environment-specific taxa support adaptation. Meanwhile, this study also has limitations, such as a small number of populations, unvalidated functions, and unclear causality between the microbiome and host adaptability. Future work should include more populations, metagenomic validation, and controlled host–plant interaction experiments to address these limitations and provide more solid theoretical and technical support for the green control of invasive pests.

## Figures and Tables

**Table 1:** Profile for sequencing and alpha diversity index of bacterial microbiota in *C. ciliata*.

Geographical Population	Sequencing Coverage	Chao1 Richness Index	ACE Richness Index	Simpson Diversity Index	Shannon Diversity Index	PD (Whole Tree)
BJ	0.9999 ± 0	173.46 ± 27.85	173.83 ± 28.33	0.67 ± 0.17	3.14 ± 0.53	19.03 ± 1.31
LYG	0.9999 ± 0	235.92 ± 85.04	235.95 ± 84.64	0.68 ± 0.25	3.31 ± 1.8	24.26 ± 3.63
NJ	0.9999 ± 0	269.04 ± 34.53	270.9 ± 33.83	0.95 ± 0.02	5.63 ± 0.35	25.41 ± 2.8

Data are presented as mean ± standard deviation (SD). BJ, Beijing population; LYG, Lianyungang population; NJ, Nanjing population.

**Table 2:** Comparison of microbial functions between three *C. ciliata* populations.

KEGG		KO Abundance (mean ± SD)			Test of Significant Difference		
Pathway ID	Description	BJ	LYG	NJ	BJ vs. LYG	BJ vs. NJ	LYG vs. NJ
K00059	3-oxoacyl-[acyl-carrier protein] reductase	214,639.89 ± 19,529.29	211,219 ± 27,538.19	286,120.5 ± 38,280.93	ns	*	*
K01992	ABC-2 type transport system permease protein	234,731.05 ± 51,539.84	205,153.58 ± 6127.21	226,240.17 ± 5289.9	ns	ns	ns
K02004	putative ABC transport system permease protein	295,834.58 ± 86,884.59	176,857.04 ± 30,439.67	187,111.23 ± 6520.6	ns	ns	ns
K02529	LacI family transcriptional regulator	205,716.88 ± 24,135.29	216,059.18 ± 6935.44	226,594.9 ± 48,681.4	ns	ns	ns
K00799	glutathione S-transferase	120,290.17 ± 42,171.62	151,199.27 ± 26,406.15	348,300.45 ± 29,615.49	ns	**	*
K01990	ABC-2 type transport system ATP-binding protein	214,214.58 ± 68,862.95	158,257.43 ± 14,476.75	182,882.15 ± 1153.82	ns	ns	ns
K02003	putative ABC transport system ATP-binding protein	205,418.03 ± 63,705.68	131,352.23 ± 77,379.26	164,448.66 ± 25,499.01	ns	ns	ns
K03704	cold shock protein (beta-ribbon, CspA family)	151,161.72 ± 3448.62	178,252.38 ± 22,863.59	170,936.02 ± 16,102.79	*	*	ns
K03406	methyl-accepting chemotaxis protein	108,609.14 ± 19,513.09	82,246.02 ± 51,772.27	291,506.89 ± 40,176.34	ns	*	*
K03832	periplasmic protein TonB	177,325.89 ± 15,478.43	142,216.32 ± 20,946.48	146,972.12 ± 43,251.28	ns	ns	ns
K02030	polar amino acid transport system substrate-binding protein	88,779.82 ± 9553.29	130,471.27 ± 25,217.59	216,552.85 ± 35,043	*	**	ns
K02029	polar amino acid transport system permease protein	66,701.83 ± 9234.3	98,198.26 ± 14,399.29	193,059.56 ± 119,790.32	*	***	*
K02035	peptide/nickel transport system substrate-binding protein	57,599.91 ± 11,109.22	101,006.41 ± 9329	187,563.72 ± 69,608.95	*	***	*
K01999	branched-chain amino acid transport system substrate-binding protein	56,494.17 ± 9553.08	81,926.94 ± 8651.07	183,807.94 ± 67,949.19	*	***	*
K02049	NitT/TauT family transport system ATP-binding protein	48,438.7 ± 18,127.01	78,344.85 ± 11,047.38	163,954.09 ± 38,445.11	*	***	*
K02033	peptide/nickel transport system permease protein	44,440.14 ± 12,442	72,340.18 ± 20,090.37	173,113.19 ± 92,535.72	ns	**	*
K02032	peptide/nickel transport system ATP-binding protein	43,487.05 ± 13,312.34	70,394.63 ± 19,760.78	169,473.54 ± 90,693.3	ns	**	*
K03559	biopolymer transport protein ExbD	85,323.53 ± 5826.76	68,159.24 ± 11,476.05	114,639.73 ± 23,758.61	*	ns	**
K01996	branched-chain amino acid transport system ATP-binding protein	48,458.08 ± 6975.92	72,372.96 ± 7737.57	143,288.47 ± 45,444.15	*	***	*
K02050	NitT/TauT family transport system permease protein	42,626.69 ± 9676.72	64,526.41 ± 795.72	156,860.58 ± 50,846.55	*	***	*

The top 10 microbial functions by abundance and the top 10 microbial functions with significant differences in abundance are displayed. Data are presented as mean ± SD. Asterisks indicate significant differences: *n* = 3; * *p* < 0.05; ** *p* < 0.01; *** *p* < 0.001; ns = non-significant.

## Data Availability

The *16S rRNA* sequencing data have been submitted and stored in the National Center for Biotechnology Information (NCBI) Sequence Read Archive (SRA) [https://www.ncbi.nlm.nih.gov/sra, accessed on 1 September 2025] with the BioProject accession number PRJNA1313396.
